# Predominance of HA-222D/G Polymorphism in Influenza A(H1N1)pdm09 Viruses Associated with Fatal and Severe Outcomes Recently Circulating in Germany

**DOI:** 10.1371/journal.pone.0057059

**Published:** 2013-02-25

**Authors:** Marianne Wedde, Stephanie Wählisch, Thorsten Wolff, Brunhilde Schweiger

**Affiliations:** Robert Koch-Institut, National Reference Centre for Influenza, Berlin, Germany; The University of Hong Kong, China

## Abstract

Influenza A(H1N1)pdm09 viruses cause sporadically very severe disease including fatal clinical outcomes associated with pneumonia, viremia and myocarditis. A mutation characterized by the substitution of aspartic acid (wild-type) to glycine at position 222 within the haemagglutinin gene (HA-D222G) was recorded during the 2009 H1N1 pandemic in Germany and other countries with significant frequency in fatal and severe cases. Additionally, A(H1N1)pdm09 viruses exhibiting the polymorphism HA-222D/G/N were detected both in the respiratory tract and in blood. Specimens from mild, fatal and severe cases were collected to study the heterogeneity of HA-222 in A(H1N1)pdm09 viruses circulating in Germany between 2009 and 2011. In order to enable rapid and large scale analysis we designed a pyrosequencing (PSQ) assay. In 2009/2010, the 222D wild-type of A(H1N1)pdm09 viruses predominated in fatal and severe outcomes. Moreover, co-circulating virus mutants exhibiting a D222G or D222E substitution (8/6%) as well as HA-222 quasispecies were identified (10%). Both the 222D/G and the 222D/G/N/V/Y polymorphisms were confirmed by TA cloning. PSQ analyses of viruses associated with mild outcomes revealed mainly the wild-type 222D and no D222G change in both seasons. However, an increase of variants with 222D/G polymorphism (60%) was characteristic for A(H1N1)pdm09 viruses causing fatal and severe cases in the season 2010/2011. Pure 222G viruses were not observed. Our results support the hypothesis that the D222G change may result from adaptation of viral receptor specificity to the lower respiratory tract. This could explain why transmission of the 222G variant is less frequent among humans. Thus, amino acid changes at HA position 222 may be the result of viral intra-host evolution leading to the generation of variants with an altered viral tropism.

## Introduction

Influenza A(H1N1)pdm09 viruses are characterized by an unique combination of gene segments. The PB2, PB1, PA, HA, NP and NS genes are similar to those previously detected in triple-reassortant swine influenza viruses circulating in pigs in North America whereas the NA and M segments are most closely related to genes of influenza A viruses found in swine in Eurasia. The genes encoding HA, NP and NS of the previous North American triple-reassortant swine influenza A (H1) virus originated from classic swine influenza A viruses, PB2 and PA genes from avian influenza viruses from the North American lineage and the PB1 gene from human influenza A viruses A(H3N2) [1], [2]. In the course of the A(H1N1)pdm09 pandemic the virus caused globally around 201,200 respiratory deaths and 83,300 cardiovascular deaths [3]. The A(H1N1)pdm09 virus has shifted into the post pandemic period since 10th of August 2010 and is still circulating worldwide. In Germany, 40,548 clinical/laboratory confirmed influenza cases were reported to the Robert Koch-Institut (RKI) from the first of October 2010 to the 15th of April 2011. Of these, 6,216 (15%) were hospitalized. Sentinels proved that the majority of confirmed influenza cases (62%) were A(H1N1)pdm09 viruses. Moreover, of 148 fatal cases 126 (85%) were attributed to an A(H1N1)pdm09 infection [4].

In the course of the A(H1N1)pdm09 pandemic a D222G (H1 numbering) substitution in the viral haemagglutinin (HA) gene was detected with significant frequency in fatal and severe cases [5], [6]. The HA protein is an antigenic surface protein and mediates both the binding of the virus to the host cell and the subsequent fusion process. The receptor binding site (RBS) of the HA protein is composed of three structural elements: a 190-helix (residues 184–191), a 220-loop (residues 218–225), and a 130-loop (residues 131–135), while other highly conserved residues, (Tyr91, Trp150, His180, and Tyr192) form the base of the pocket [7]. Parts of the RBS represent antigenic sites, as shown for the conserved amino acid (aa) 192 and aa 184–191 in the 190-helix (Sb) and aa 222 in the 220-loop (Ca2) [8]. The receptor-binding specificity of avian and human influenza viruses is defined by the aa exposed in the HA receptor-binding pocket. Human influenza viruses preferably attach to sialic acid that is linked to galactose by an α2,6-linkage (SAα2,6Gal) which is found on human epithelial cells in nasal mucosa, paranasal sinuses, pharynx, trachea and bronchi. In comparison, avian influenza viruses preferentially bind to SAα2,3Gal expressed on epithelial cells in the intestinal tract of waterfowl [9], [10].

A hypothesis suggests that the virus causing the Spanish Flu A(H1N1) in 1918 was able to cross the species barrier between birds and humans by mutations in the HA which changed the binding preference from the avian to the human form [11]. Comparison of the avian HA consensus sequence with HA sequences from the 1918 influenza virus demonstrates that only one or two of the conserved residues (187, 222), depending on the viral isolate, are different [12]. Accordingly, for H1 HAs it could be shown that aa at positions 187 and 222 define the receptor-binding specificity. HA-187D and HA-222D lead to the binding of human-type receptors, whereas 187E and 222G induce binding to avian-type receptors [9], [13]. In contrast to earlier assumptions, these avian-type receptors are not restricted to birds. They are also located on human epithelial cells lining the respiratory bronchiole and the alveolar walls and could, therefore, enable highly pathogenic viruses like A(H5N1) to replicate in the lower respiratory tract [9], [10].

In the current study, we focused particularly on the residue 222 representing a major determinant of HA receptor specificity in H1 HAs because mutations at position 222 could potentially change viral tropism and may lead to greater intra-host evolution. We report on the analysis of the residue 222 within the HA gene of A(H1N1)pdm09 viruses using the PSQ technique and show that the prevalence of HA-222 quasispecies increased from 2009/2010 to 2010/2011, from 10.2% to 60%.

## Materials and Methods

### Ethics Statement

The clinical samples were taken from patients exhibiting influenza-like illness (ILI) who gave verbal consent for laboratory examination. The verbal consent was not documented. Samples were sent to the National Reference Centre (NRC) for Influenza at the RKI (Berlin, Germany) for influenza virus surveillance purposes in Germany. Specimens from patients with mild respiratory infections were taken by sentinel practitioners, samples from severe and fatal respiratory cases were collected by the attending physicians in hospitals. The medical practices were located all over Germany. The analyses of all data were done anonymously. Consequently, ethic committee approval was not required since such a sentinel surveillance is covered by German legislation (§13, §14, Protection against Infection Act).

### Clinical samples

Human nasopharyngeal aspirates, nasal or throat swabs (upper airway) as well as tracheal or bronchial secretions, bronchoalveolar lavages (BAL), lung (lower airway) or heart tissues and RNA or cDNA were analyzed that were collected in the seasons 2009/2010 and 2010/2011 (from October until April). The clinical outcome (mild, severe or fatal) was classified according to World Health Organization (WHO) guidance criteria [14]. Mild influenza is associated with typical ILI symptoms, including sudden onset, fever, cough, sore throat, rhinorrhoea, headache, muscle pain and malaise, but no shortness of breath and no dyspnoea. Patients may present with some or all of these symptoms. Severe influenza is associated with clinical (e.g. shortness of breath/dyspnoea, tachypnea and/or hypoxia) and/or radiological signs of lower respiratory tract disease (e.g. pneumonia) or other complications (e.g. myocarditis). For more detailed information please refer to WHO guidance criteria [14] and Table 1. Specimens from mild cases were collected from the upper airway, and specimens from fatal and severe cases were collected from both the upper and the lower airways. Swabs were transported in 1 ml UTM-RT medium (Copan, Murrieta, USA) and upon arrival adjusted to 3 ml with sterile minimal essential medium with HEPES (Gibco BRL, Eggenstein, Germany) and 100 U/ml penicillin-streptomycin (Gibco) and aliquoted. For cell culturing, an aliquot of each specimen was filtered (0.2 µm). Specimens were stored at −70°C. Other specimens, like nasopharyngeal aspirates, tracheal or bronchial secretions as well as BAL were used undiluted. Viscous samples were liquefied by N-acetyl-cysteine.

**Table 1:** Clinical characteristics of patients with influenza A(H1N1)pdm09 virus infection associated with fatal and severe outcomes.

Characteristic	Patients A(H1N1)pdm09 PCR-positive (n = 55) in 2009/2010	Patients A(H1N1)pdm09 PCR-positive (n = 52) in 2010/2011
Age in median years (range)	39 (0–73)	38 (0–66)
Male	25	28
Female	21	20
Underlying illness (e.g. example asthma, diabetes):	16	11
Including immunosuppressed patients and patients with leukemia	6	7
Pneumonia, acute respiratory distress syndrome (ARDS), respiratory insufficiency	9	24(*including 9 sentinel samples)
Intensive care unit (ICU), ventilation	7	5
Myocarditis/encephalitis symptom	3	2
Fatal cases	26	15

The characteristics were indicated as provided by the attending physicians.

* In 2009/2010 and 2010/2011 the A(H1N1)pdm09 PCR-positive samples associated with fatal and severe outcomes which were provided by hospitals were analyzed in the current study. Additionally, in 2010/2011 all sentinel samples sent by the medical practices were screened and the A(H1N1)pdm09 PCR-positive specimens that were associated with pneumonia were included in the group of severe outcomes.

### Viruses and bacteria

Influenza A(H1N1)pdm09 viruses were obtained from the strain collection of the NRC Influenza at RKI and were collected during 2009/2010 and 2010/2011. Enteroviruses were provided by S. Diedrich (NRC for Poliomyelitis and Enteroviruses at RKI). Respiratory bacteria like *Streptococcus pneumoniae* were provided by M. van der Linden (NRC for Streptococci at the Institute for Medical Microbiology, University Hospital, Aachen, Germany) and Staphylococcus strains by W. Witte (NRC for Staphylococci at RKI).

### RNA extraction and cDNA synthesis

Viral RNA were extracted using 400 µl diluted specimen and the MagAttract Viral RNA Kit M48 (Qiagen, Hilden, Germany) or the RTP DNA/RNA Virus Mini Kit (Invitek, Berlin, Germany). Tissue samples from heart and lung were thoroughly crushed prior RNA extraction. cDNA synthesis was performed using 25 µl RNA and 15 µl reaction mixture containing dithiothreitol (DTT, 2.5 mM, Invitrogen, Karlsruhe, Germany), deoxynucleotide triphosphates (dNTPs, each of 200 µM, Invitrogen), random hexamer primers (500 nM, Metabion, Martinsried, Germany), 1 U RNasin (Promega, Madison, WI) and 5 U moloney murine leukemia virus (M-MLV) reverse transcriptase in first-strand buffer (Invitrogen). RT reaction was performed under the following conditions: 42°C (5 min), 37°C (30 min) and 95°C (5 min). Extraction control and identification of A(H1N1)pdm09 virus-positive samples were done by real-time PCR of HA, NA and M segments [15].

### A(H1N1)pdm09 virus-specific RT-PCR

A(H1N1)pdm09 virus-specific RT-PCRs were performed with a PE 9700 thermal cycler (Perkin Elmer, Massachusetts, USA) or Eppendorf thermal cycler (Eppendorf AG, Hamburg, Germany) using primer listed in Table 2. Each PCR was performed using 5 µl cDNA per 50 µl reaction volume containing MgCl_2_ (2 mM, Invitrogen), dNTPs (each of 200 µM, Invitrogen), primer forward/reverse (250 nM, Tib Molbiol, Berlin, Germany) and 1 U PlatinumTaq in PCR buffer (Invitrogen) under the following conditions: HAsw-F554/R768: 94°C (5 min), 45 cycles 94°C (15 sec), 54°C (30 sec), 72°C (30 sec) and finally 72°C (5 min). To confirm amplification, PCR products were analyzed on a 2% agarose gel and visualized by ethidium bromide staining under UV illumination. The amount of PCR products was quantified with the appropriate software (Herolab, Wiesloch, Germany).

**Table 2:** PCR and PSQ assays designed for characterization of influenza A(H1N1)pdm09 virus.

Seg-ment	PCR primer	Sequence	Ampli-con position (nt)^A^	Ampli-con length (bp)	PSQ primer	Sequence	Target posi-tion (nt)^A^	Target posi-tion (aa)^B^
HA	HAsw-F554	5′- GTGCTGACCAACAAAGTCTCT-3′	554–768	215	SF662^C^	5′- CAATAAGACCCAAAGTGAG-3′	664–666	222
	HAsw-R768	5′ biotin- TGCGAATGCATATCTCGG-3′			SF662a^D^	5′- CAATAAGACCYAAAGTGAG-3′		
					SF663^D^	5′- CAATAAGACCYAAAGTGAGG-3′		

A Nucleotide numbering of HA is relative to the first codon after the signal sequence.

B Aa numbering of HA is relative to the H1 mature protein of A/California/07/2009.

C Primer was used for PSQ analysis of viral HA gene in 2009/2010 with a cyclic dispensation order (ACGT)_10_ in SQA mode (SF662).

D Primer were used for PSQ analysis of viral HA gene in 2010/2011 with a cyclic dispensation order (ACGT)_10_ in SQA mode (SF662a, SF663) and a customized dispensation order ATATGTAT(ACGT)_5_ (SF663). The PSQ primer SF663 was also described recently [39].

### PSQ

About 30 µl of biotinylated PCR fragment (<300 bp) were immobilized onto 4 µl Streptavidin-Sepharose (Healthcare Bio-Sciences AB, Uppsala, Sweden) including binding buffer (Biotage/Qiagen) by incubation at room temperature for 5 min, followed by additional 5 min at room temperature with agitation at 1,400 rpm. Single-stranded DNA was obtained by washing the immobilized PCR product with 70% EtOH, denatured with 0.2 M NaOH, and washed with washing buffer (Biotage/Qiagen), using a Vacuum Prep Tool and Vacuum Prep Worktable (Biotage/Qiagen). The immobilized single-stranded DNA was then suspended in 40 µl annealing buffer (Biotage/Qiagen) containing 40 pmol sequencing primer and hybridized to the sequencing primer by incubation at 80°C for 2 min and at room temperature for 5 min. Primed DNA was sequenced using the PyroMark ID system (Biotage/Qiagen). Sequencing was performed in a total volume of 40 µl using 5×96 PyroMark ID Pyro Gold Reagent (Biotage) and specific cyclic sequence analysis (SQA) entry for each PSQ of *de novo* sequence strategy. Negative-control nucleotide samples were included to measure background signals (controls recommended by Biotage/Qiagen). Raw data were analyzed with BioEdit using reference sequences for alignment. Sequencing primer and target sequences are listed in Table 2.

### A(H1N1)pdm09 virus HA-specific quantitative real-time PCR

The amount of HA genome copies in A(H1N1)pdm09 virus specimens was analyzed by pandemic H1-specific real-time PCR using the Light Cycler 480 (Roche, Basel, Switzerland). A standard curve was created by three times of serial dilution (10^6^–10^0^ copies/PCR) of synthetic H1 plasmid. Real-time PCR was performed using 3–5 µl cDNA or plasmid and a reaction mixture including MgCl_2_ (5 mM, Invitrogen), dNTPs (each of 200 µM, Invitrogen) containing dUTPs (GE Healthcare), primer FluSw H1 F236 (300 nM), primer FluSw H1 R318 (300 nM), FluSw H1 probe TM292 (100 nM) and 0.5 U Platinum® Taq DNA Polymerase in PCR buffer (Invitrogen). Finally, the PCR mixture was adjusted to a total volume of 25 µl with H_2_O and the real-time PCR was carried out under the following conditions: 95°C (5 min), 45 cycles of 95°C (15 sec), 60°C (30 sec) [15].

### Cycle sequencing

PCR products of amplified HA were sequenced by automated nucleotide cycle sequencing (primer sequence on request). PCR products were first purified using an MSB® Spin PCRapace Kit (Invitek) and then sequenced using the BigDye® Terminator v3.1 Cycle Sequencing Kit (Applied Biosystems, Darmstadt, Germany) and a capillary sequencer 3130xl (Applied Biosystems).

### TA Cloning

PCR products of amplified HA genes were cloned using the TOPO TA Cloning® Kit (Invitrogen). Freshly produced amplicons (with single 3′-deoxyadenosine [A] overhangs) were ligated into the pCR®2.1-TOPO® vector (insert∶vector = 3∶1) according to the protocol for chemically competent *E. coli*. Following the cloning reaction, Top10-competent cells were transformed using the One Shot® chemical transformation protocol. The clones were selected on ampicillin-containing culture plates and were subsequently selected by blue/white screening.

### Development of the HA-222 PSQ-PCR assay

PCR and PSQ primers were designed to analyze the aa 222 on the HA gene of A(H1N1)pdm09 viruses. Viral RNA sequences were acquired from the Global initiative on sharing all influenza data (GISAID), Genbank at the National Centre for Biotechnology Information (NCBI) and NRC for Influenza at the RKI, Germany, and aligned with Bio Edit (7.0.9.0) and MEGA 4.0.2. Specific RT-PCR and PSQ primers were designed with PyroMark Assay Design Software 2.0 (Biotage AB, Uppsala, Sweden). The designed primers (Table 2) were evaluated for A(H1N1)pdm09 virus specificity by BLAST (NCBI) and PCR methods. Blast search revealed that the PCR-PSQ primer were highly specific for A(H1N1)pdm09 viruses which was confirmed by PCR. Influenza A and B, other respiratory viruses and bacteria were prepared for determination of cross-reactivity of designed A(H1N1)pdm09 virus HA-222 PCR. No cross-reactivity of designed primers either with other influenza A subtypes and type B viruses or other respiratory viruses and bacteria was observed. The sensitivity of the designed HA-222-PCR was determined using 15 A(H1N1)pdm09 PCR-positive, randomly selected specimens containing different viral loads (CT 20–37). A positive result was obtained for all specimens that had real-time PCR CT values of 35 or lower. Thus, the detection limit of the designed PCR assay was about ten genome copies. The sensitivity of the HA-222 PSQ-PCR assay was determined using A(H1N1)pdm09 variants circulating in 2009/2010 and 2010/2011 and was evaluated on at least two separate occasions. The concentration of the genome copies in the viral cDNAs was determined by pandemic H1-specific real-time PCR. The viral cDNAs were serially diluted to final concentrations from 10^6^ to 10^0^ genome copies per assay and the HA-222 target was amplified and pyrosequenced. The PSQ analysis revealed that about ten genome copies were provable. To determine the sensitivity of the assay for detection of HA-222D/G polymorphism, 222D and 222G variants were amplified by the designed HA-PCR and TA cloned. Subsequently, the clones were serially diluted to final concentration from 10^6^ to 10^1^ genome copies per PCR assay, adjusted to defined 222D/G mixtures (100/0, 95/5, 90/10, 80/20, 50/50, 20/80, 10/90, 5/95, 0/100%) and were amplified and pyrosequenced. The sensitivity of the PSQ-PCR was evaluated on at least two separate occasions and was carried out twice. The evaluation of the pyrograms revealed that the assay for identification of HA-222 variants in mixtures was linearly from 10^6^ (maximum tested) to 10^3^ genome copies (approximately CT 18–29). In this range the detection limit of minor variants was about 10%.

## Results

### PSQ analysis of the aa 222 on HA gene

**Figure 1:**
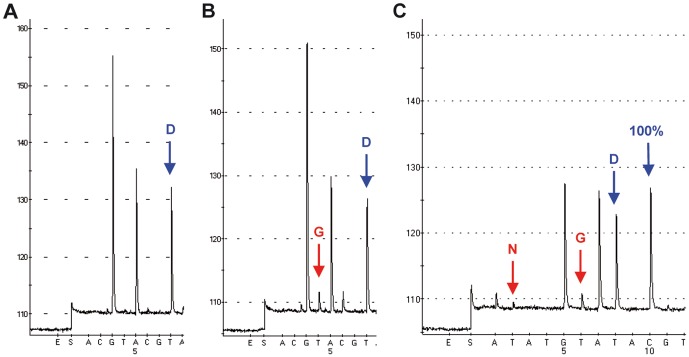
Detection of pure and heterogeneous HA-222 variants using the cyclic dispensation order (ACGT)_10_ and the customized dispensation order ATATGTAT(ACGT)_5_. The pyrograms represent the wild-type HA-222D (GGAT) using the PSQ primer SF662 and (ACGT)_10_ (A), HA-222D/G (GGAT/GGGT) polymorphism of a fatal case (448/11) utilizing the PSQ primer SF662A and (ACGT)_10_ (B) and HA-222D/G/N polymorphism of the same specimen (448/11) as in (B) applying the PSQ primer SF663 and ATATGTAT(ACGT)_5_ (C).

To analyse the residue 222 within the HA gene of A(H1N1)pdm09 viruses specimens from mild (n = 159/99) and fatal and severe (n = 49/50) cases were collected in the 2009/2010 and 2010/2011 seasons, respectively. For rapid and large scale analysis of the polymorphic marker HA-222 a PSQ-PCR assay was designed (Table 2). A cyclic dispensation order (ACGT)_10_ was used for HA-222 analysis in cases from the season 2009/2010 resulting in the detection of HA-222 variants, as shown for HA-222D (Fig. 1A). The identified HA-222 quasispecies have to be confirmed by TA cloning and subsequent PSQ. To avoid the time consuming cloning procedure, the PSQ assay has been further developed to allow for a more detailed analysis. In a first step, the standard cyclic dispensation order (ACGT)_10_ was applied which is suitable and sensitive for detection of pure variants and mixtures of those variants (Figure 1B). In a second step, the customized dispensation order ATATGTAT(ACGT)_5_ was used (Figure 1C). This customized entry enabled successively the identification of the aa (codons) 222N (AAT), 222Y (TAT), 222G (GGT), 222V (GTT), 222E (GAA) and 222D (GAT).

**Figure 2:**
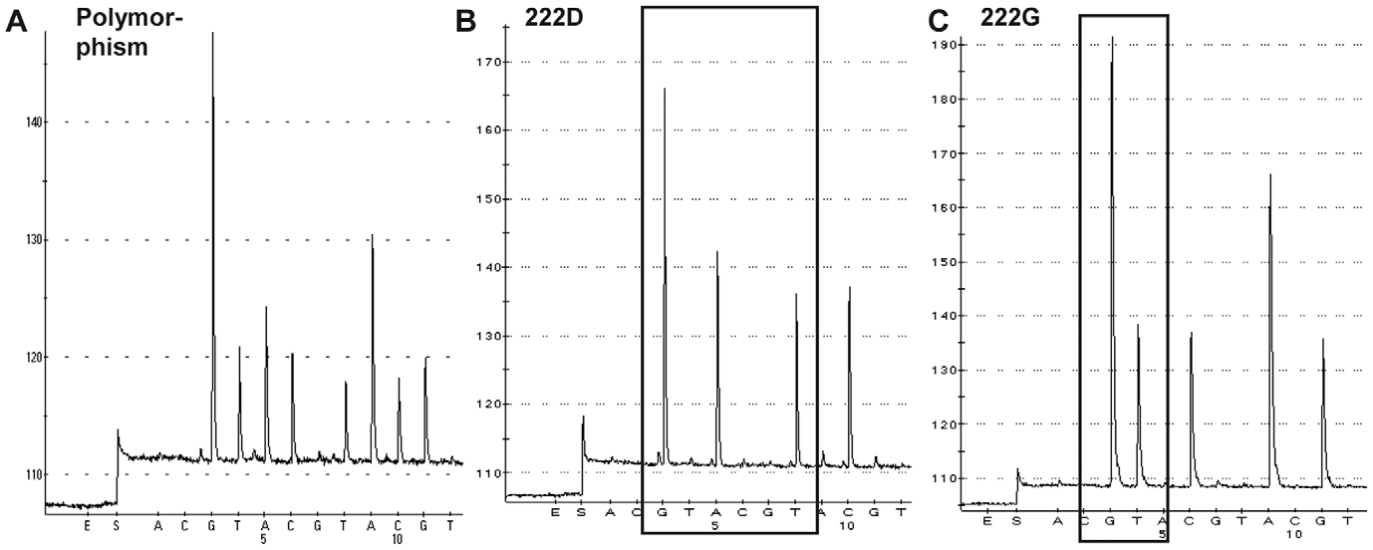
PSQ analysis of a HA-222D/G polymorphism detected in a severe case. The pyrogram shows a HA-222 polymorphism (specimen 3004/10) (A). After amplification and TA cloning of the HA-222 fragment, 30 clons were picked and subsequently pyrosequenced using the cyclic entry (CTGA)_10_. Fifteen clones are identified as wild-type 222D with codon GGAT (B) and 15 clones as variant 222G with codon GGGT (C).

**Figure 3:**
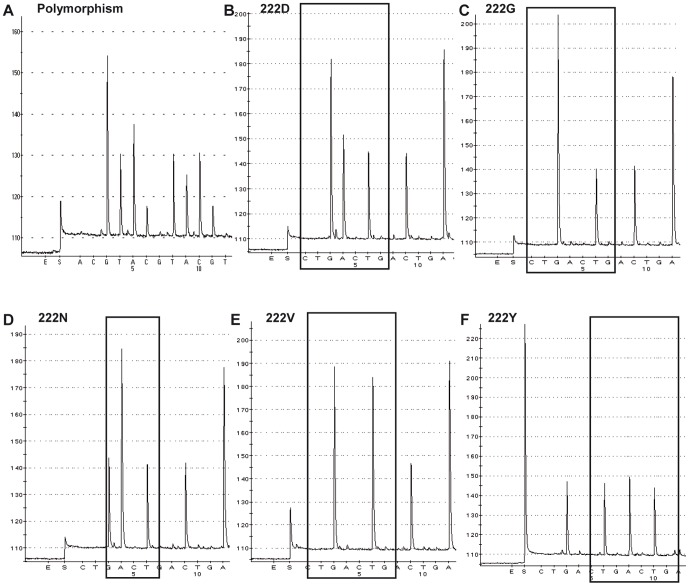
PSQ analysis of a HA-222D/G/N/V/Y polymorphism identified in a fatal case. The pyrogram represents a HA-222 polymorphism (specimen 3070/10) (A). After amplification and TA cloning of the HA-222 fragment, 23 clones were analyzed using the cyclic entry (CTGA)_10_. Three of them show the wild-type 222D with codon GGAT (B), seven 222G (GGGT) (C), four 222N (GAAT) (D), one 222V (GGTT) (E) and eight clones 222Y (GTAT) (F).

PSQ analysis revealed for 2009/2010 that out of 159 mild cases, 13 (8.2%) showed a D222E substitution, one (0.6%) a D222N change and 145 (91.2%) represented the wild-type 222D. Out of 49 severe including 24 fatal cases, three (6.1%) possessed 222E and four (8.2%) exhibited 222G. Furthermore, five (10.2%) heterogeneous HA-222 specimens were identified, suggesting that the tested samples contained different virus variants. These heterogeneous populations were found in one severe and four fatal cases, but not in mild infections (Table 3). TA cloning and PSQ as well as cycle sequencing analysis of extracted viral nucleic acids confirmed the presence of a 222D/G heterogeneity in a severe case (Fig. 2) and a 222D/G/N/V/Y polymorphism in a fatal case (Fig. 3).

**Table 3:** Detection of co-circulating HA-222 variants of influenza A(H1N1)pdm09 viruses identified during 2009/2010 and 2010/2011 by PSQ analysis.

	2009/2010								2010/2011							
HA-222^A^	Mild cases		Fatal cases			Fatal and severe cases			Mild cases		Fatal cases			Fatal and severe cases		
	%	n	%	n	P	%	n	P	%	n	%	n	P	%	n	P
**D**	91.2	145	70.8	17	0.009	75.5	37	0.006	98	97	13.3	2	<0.001	36	18	<0.001
**E**	8.2	13	8.3	2	1	6.1	3	0.768	1	1	0	0	1	0	0	1
**G**	0	0	4.2	1	0.131	8.2	4	0.003	0	0	0	0	NA	0	0	NA
**N**	0.6	1	0	0	1	0	0	1	1	1	0	0	1	0	0	1
**D/G**									0	0	73.3	11	<0.001	60	30	<0.001
**D/G/N**									0	0	13.3	2	0.016	4	2	0.111
**PM** B	0	0	16.7	4	<0.001	10.2	5	<0.001								
**Total**	100	159	100	24		100	49		100	99	100	15		100	50	

NA: not applicable.

P: p-value of difference of fatal/fatal and severe versus mild cases with HA-222 mutation, calculated by Fisher's exact test, two-sided.

A For identification of HA-222 variants, RNA was extracted from patient specimens. Mild cases collected in 2009/2010 constituted an exception, because only 10% were represented by patient specimens and 90% by cell culture isolates, respectively.

B PM: polymorphism. In 2009/2010, HA-222 polymorphisms were detected using the PSQ primer SF662 and the cyclic entry (ACGT)_10_. All HA-222 variants in mixtures could not be clearly identified by the applied cyclic entry. Two quasispecies (222D/G, 222D/G/N/V/Y) could be confirmed by TA cloning and subsequent pyrosequencing. In 2010/2011, direct pyrosequencing of all circulating 222 variants and quasispecies could be realized by the application of the PSQ primer SF663 and the customized entry ATATGTAT(ACGT)_5_.

Similar to the previous influenza season, comparable results were obtained for A(H1N1)pdm09 viruses derived from mild cases during 2010/2011 since the majority (98%) represented the wild-type 222D. One out of 99 mild cases showed a D222E and one a D222N substitution (each 1%). However, the patterns for HA-222 markers in fatal and severe cases changed strikingly in 2010/2011. The majority of A(H1N1)pdm09 viruses derived from those cases showed a heterogeneous expression of HA-222 since 30 (60%) had a 222D/G polymorphism, two (4%) expressed 222D/G/N and 18 (36%) corresponded to the wild-type 222D (Table 3). Moreover, the 222D/G polymorphism was detected more frequently in fatal (73%) and hospitalized (59%) cases than in community patients exhibiting pneumonia (33%, data not shown). Pure 222G variants were not identified in this season.

We were also interested to elucidate whether the age distribution of patients infected with the distinct HA-222 variants varied in both seasons. The analysis of 48 specimens from fatal and severe cases collected in 2010/2011 revealed that the median age of patients infected with the 222D wild-type was 38 and the median age of patients infected with the 222D/G quasispecies was 39 years, respectively. In contrast, PSQ data of 39 specimens from fatal and severe cases collected in 2009/2010 showed that the wild-type was more frequently identified in younger patients (median age 22 years) whereas the 222G variant and 222 polymorphisms were more prevalent in older patients (median age 52 years). In both seasons, the median age of patients with mild outcomes was 12 and 10 years, respectively (Table 4).

**Table 4:** Age distribution of patients infected with different HA-222 variants or HA-222 quasispecies.

	Age of mild cases			Age of fatal and severe cases		
2009/2010	Median	Range	N	Median	Range	N
D	12	1–84	122	22	0–75	32
G, D/G, PM				52	0–65	6
D/G/N/V/Y				71	-	1
Total			122			39
**2010/2011**						
D	10	0–57	94	38	1–67	15
D/G				39	0–66	31
D/G/N				41	21–61	2
Total			94			48

### Emergence, prevalence and tissue distribution of the HA-222 quasispecies

**Figure 4:**
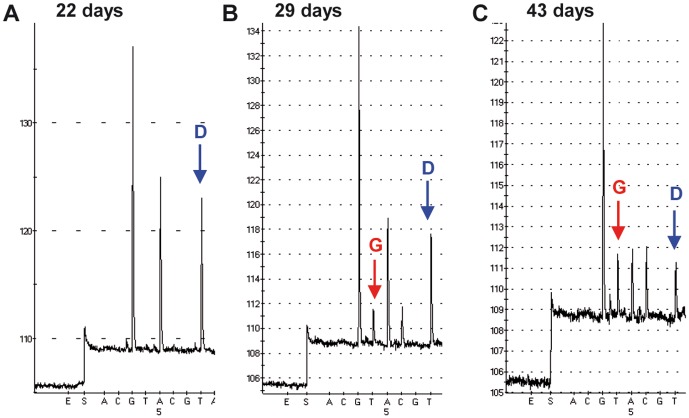
Emergence of the HA-222D/G quasispecies during a severe course of disease. The BAL samples were collected 22, 29 and 43 days after the onset of ILI symptoms (specimens 3340/11, 3341/11, 3343/11). The pyrograms show the wild-type 222D (GAT) (A), the emergence of the 222G variant (B) and the increase of the 222G variant (C) relative to the wild-type 222D.

To investigate the emergence of the HA-222 quasispecies in the course of an A(H1N1)pdm09 virus infection, five specimens obtained from a patient with a severe course of disease were analyzed. The 48-year-old man was hospitalized, immune suppressed and received extracorporeal membrane oxygenation (ECMO) as well as Zanamivir and Oseltamivir therapy. The antiviral treatment started 11 days after onset of ILI symptoms. Specimens were collected from the lower respiratory tract (BAL) within 50 days after the onset of ILI symptoms. Real-time PCR analysis showed that the total amount of genome copies decreased between days 16 and 50 after the onset of ILI symptoms by an about one thousand-fold (d16 (CT 28), d22 (CT 28), d29 (CT 32), d36 (CT 34), d43 (CT 38) and d50 (PCR negative). The PSQ analysis revealed that the wild-type 222D was predominant in the initial phase of the disease but declined with its progression. Twenty-nine days after the onset of ILI symptoms the 222G variant appeared and the 222D/G quasispecies was detectable until day 43. During this time period the proportion of the 222G variant increased relative to the wild type 222D (Fig. 4).

Eighteen fatal and severe cases from 2010/2011 were further analyzed to elucidate putative correlations between the HA-222 polymorphism and the duration of the disease. Generally, each specimen represented one single time point. This analysis lead to the detection of 222D/G polymorphisms in later phases of infection and the median time after onset of ILI symptoms was four days. In contrast, the 222D wild-type was identified in an earlier phase of infection. The median time after onset of ILI symptoms was one day which is comparable with the group of mild cases infected with the 222D wild-type. Five of the nine severe cases that were infected with the 222D virus represented community patients associated with pneumonia (median time one day). The remaining four patients were hospitalized (median time three days). In contrast, in the panel of samples collected in 2009/2010, the 222D wild-type was identified in later phases of disease (median time five days) mainly in hospitalized patients with a severe outcome. In this context, no data were available for the group of severe cases associated with the D222G change in 2009/2010 (Table 5).

**Table 5:** Time after onset of ILI symptoms and occurrence of the HA-222G variant or HA-222 quasispecies.

	Days after onset of ILI-symptoms											
	Mild cases			Fatal and severe cases								
	Community patients			ACommunity patients			Hospitalized patients			ACommunity and hospitalized patients		
2009/2010	Median	Range	N	Median	Range	N	Median	Range	N	Median	Range	N
D	1	0–4	119				5	1–12	4	5	1–12	4
G, D/G, PM												
D/G/N/V/Y												
Total			119									4
**2010/2011**												
D	1	0–5	91	1	0–4	5	3	0–7	4	1	0–7	9
D/G				4	-	2	4	2–5	7	4	2–5	9
D/G/N												
Total			91									18

The specimens of the 48-year-old hospitalized man that were collected within 50 days after onset of ILI symptoms were not included in the analysis. Generally, each specimen represented one single time point.

A The community patients associated with pneumonia were included in the group of fatal and severe cases.

To determine the prevalence of the D222G substitution and the 222D/G quasispecies in different compartments of the respiratory tract, upper and lower airway samples received from fatal and severe cases in 2009/2010 and 2010/2011 were analyzed. In general, each specimen represented one patient, because paired upper and lower airway specimens were rarely received. PSQ analysis showed that in the seasons 2009/2010 and 2010/2011 the 222G variant or 222 quasispecies were detectable in upper respiratory tract samples (15.4% and 57.9%, respectively) as well as with slightly higher prevalence in the lower respiratory tract (18.8% and 64.7%, respectively; Table 6). Interestingly, the analysis of the 222D/G mixtures identified in 2010/2011 revealed that the proportion of 222G was generally higher in the lower than in the upper airway (data not shown).

**Table 6:** Prevalence of co-circulating HA-222 variants and HA-222 quasispecies of influenza A(H1N1)pdm09 viruses in the upper and lower respiratory tract identified in 2009/2010 and 2010/2011.

	Mild cases - upper respiratory tract		Fatal and severe cases - upper respiratory tract		Fatal and severe cases -lower respiratory tract	
2009/2010	%	n	%	n	%	n
D	100.0	122	84.6	11	75.0	12
**G, D/G, PM**			15.4	2	18.8	3
D/G/N/V/Y					6.3	1
Total	100.0	122	100.0	13	100.0	16
**2010/2011**						
D	100.0	94	42.1	8	29.4	5
D/G			57.9	11	64.7	11
D/G/N					5.9	1
Total	100.0	94	100.0	19	100.0	17

Data represented mainly single upper or lower airway specimens of one patient.

**Figure 5:**
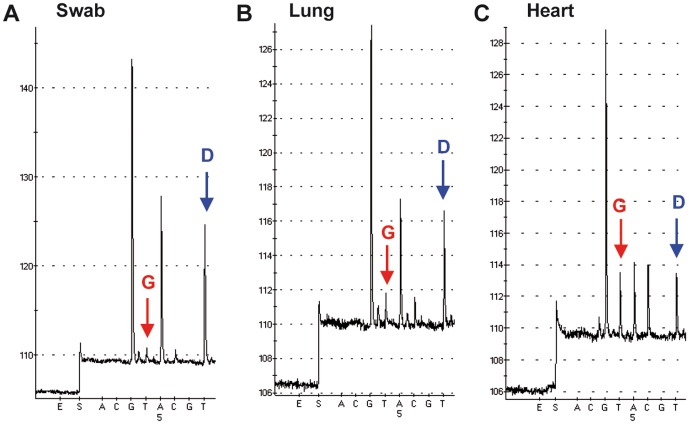
Tissue distribution of the HA-222D/G quasispecies associated with a fatal clinical outcome. A nasal swab, lung and heart tissue samples were collected (specimens 2158–2160/11). The pyrograms demonstrate a 222D/G (GAT/GGT) polymorphism in the nasal swab (A), an increase of 222G in the lung (B) and in the heart tissue (C) relative to the wild type 222D.

To study further the tissue distribution of the HA-222 quasispecies within a patient at one time-point, three specimens collected in 2010/2011 from a patient with a fatal outcome were analyzed. The 10-year-old boy had received intensive care and mechanical ventilation prior to its death. Specimens were taken from the upper airway (nasal swab), from the lower airway (lung tissue) and from the heart tissue. PSQ analysis showed that the wild-type 222D predominated in the nasal swab. However, the proportion of the 222G variant was increased slightly in the lung and profoundly in the heart tissue (Fig. 5). Notably, the total amount of HA genome copies, in the nasal swab sample (CT 29) was between hundred and thousand times higher than in the lung (CT 36) or in the heart tissue (CT 39), respectively, as quantified by real-time PCR.

## Discussion

In this study we analyzed the HA-222 polymorphism in influenza A(H1N1)pdm09 viruses associated with fatal and severe clinical outcomes in Germany since October 2009. Studies from Norway, Hong Kong and Scotland had shown that the D222G change was detectable in viruses obtained from fatal and severe cases and ranged in this group between 4 and 18% [5], [6], [16], [17]. Moreover, the European Centre for Disease Prevention and Control [18] and the World Health Organisation [19] reported on the appearance of 222G variants in 20 other countries, occasionally also in mild cases. Accordingly, the D222G mutation was identified in some mild cases (0.9%) but predominated in fatal and severe cases (5.8%) in Italy [20].

Evidence has been provided that a H1N1 virus variant (A/New York/1/18) circulating during the Spanish Flu 1918 also expressed HA-222G [11] which binds to both human SAα2,6Gal- and avian SAα2,3Gal-receptors [21]. Recently, similar receptor specificity was shown for 222G variants of A(H1N1)pdm09 virus [22], [23]. These findings required further studies since SAα2,3Gal-receptors were identified in the lower respiratory tract of humans and thus 222G variants might course a more severe disease [10]. Studies in a ferret model showed that the HA wild-type of the recombinant 1918 virus with the 187D/222D constellation conferred efficient transmission between animals, but that the HA-187D/222G mutant was inefficiently transmitted [24]. Interestingly, decreased transmission was not observed for the 222G variant of the A(H1N1)pdm09 virus, since the introduced D222G substitution had no detectable effect on transmission in animal models tested [23].

During the pandemic in 2009/2010, the D222G substitution described here was associated with fatal and severe clinical outcomes. Moreover, the wild-type 222D and the variant 222G could be identified in a specimen obtained from a severe case. This observation was in accordance with other studies reporting on 222G and 222N viruses and sometimes mixtures thereof [5], [6], [16]–[18], [25]. Furthermore, A(H1N1)pdm09 viruses identified between May and July 2009 in Australia showed also a 222D/G/N/S polymorphism. This heterogeneity was observed in virus specimens from severe cases but was also found in those from mild cases [26]. Here we describe for the first time the identification of a 222D/G/N/V/Y polymorphism in the lower airway of a fatal case. Analysis of HA sequences submitted to Genbank from April 2009 to May 2010 revealed no similar HA heterogeneity. The emergence of such diverse polymorphism of HA-222 (222D/G/N/V/Y) might be explained by adaptation of viral receptor specificity to the lower respiratory tract in the course of viral evolution in humans. Accordingly, as reported here and in a study from China [25] the HA-222 polymorphism was more frequently detected in the lower airway of A(H1N1)pdm09 infected patients. As shown in this study, high variability of HA-222 (one case of 222D/G/N/V/Y and two cases of 222D/G/N polymorphism) were exclusively identified in fatal cases. Further studies will be required to determine the relevance of this heterogeneity in position 222 of the HA for viral receptor specificity and pathogenicity.

In the post-pandemic period during 2010/2011, an increase of heterogeneous HA-222 viruses from fatal and severe cases was observed in Germany since 60% exhibited a 222D/G polymorphism. However, almost all viruses from mild cases displayed the wild-type HA-222D. Controversial results were reported from England [27] demonstrating that viruses from fatal and non-fatal outcomes, circulating in 2010/2011, generally had 222D in the HA gene. In contrast, a study from Italy showed that 222G/N variants were more frequently detected in patients admitted to the intensive-care unit for invasive mechanical ventilation or extracorporeal membrane oxygenation than in patients hospitalized in other units and community patients [28], [29]. Accordingly, the 222D/G polymorphism described here was more abundant in the fatal and in the hospitalized cases than in the community patients.

Regarding sampling time and the course of disease it should be noted that the specimens of community patients were mostly collected earlier after the onset of ILI symptoms than those of hospitalized patients, which are usually analyzed later after their admission to the clinics. In severe and fatal cases from 2010/2011, the HA-222 polymorphism was more abundant in later phases of infection and was rarely present in earlier phases suggesting that the D222G substitution occurred upon intra-host evolution. Interestingly, no D222G change was detected in specimens from hospitalized younger patients (median age 22 years) during 2009/2010, although the patients were infected for several days with the 222D variant. However, the HA-222 polymorphism predominated in older patients (median age 52 and 39) in both seasons. Thus, the marked difference concerning the prevalence of the 222D/G polymorphism between the two seasons analyzed might be attributed to the fact that in 2009/2010 frequently children that were not susceptible for viral residue 222 evolution experienced a severe outcome. In 2010/2011, predominantly adults developing a severe outcome were susceptible for the polymorphism at HA position 222.

Our data suggest that viruses with a D222G substitution exhibit an altered tropism which accompanies viral progression into the lower airways. However, at present it remains unclear whether the appearance of these variant viruses is a cause or rather a consequence of more severe disease. It should be noted that the impact of viral tropism for the pathogenicity of influenza A viruses is controversially discussed. A study using a human lung model indicated that differences in the pathogenic potential of A(H1N1)pdm09 and highly pathogenic avian A(H5N1) viruses cannot be attributed to a distinct cellular tropism alone, but is rather associated with the capacity to replicate in the type II pneumocytes and to overcome the induced cytokine response [30]. In contrast, binding studies with A(H1N1)pdm09 viruses showed that the D222G mutant with dual receptor specificity exhibited increased binding capacity to ciliated epithelial cells, which are prominent along the entire airway epithelium, as well as to type II pneumocytes and alveolar macrophages, respectively. Thus, the 222G variant may have the potential to interfere with the mucociliary clearance function and to cause more severe pulmonary infections [22], [23].

Here, we demonstrated in a severe A(H1N1)pdm09 infection that the prevalence of the predominant 222D wild-type decreased, while there was a relative increase in the proportion of the 222G variant during the course of infection. Moreover, the analysis of a fatal case identified the 222D/G polymorphism in the upper and lower respiratory tract as well as in the heart tissue with increasing prevalence in the latter ones. These observations suggest a scenario in which the wild-type virus dominates in the initial phase of infection associated with high viral loads in the upper respiratory airway. In the advanced phase of a severe infection also the lower tract becomes involved and the 222G variant may evolve by adaptation of viral receptor specificity to cells in the lower airway. The 222D/G polymorphic viruses may differ in their efficacy to replicate in the type II pneumocytes, as shown in human respiratory cells *in vitro* [31]. Here, we show that the proportion of 222G increased relative to 222D. Infection of the type II pneumocytes might result in an impaired cell function including disrupted repair of the epithelium after alveolar damage [23]. As a consequence, viremia occurs and the 222D/G viruses may be transported via the bloodstream to other organs in which the replication levels are low but sufficiently high to cause inflammation. Accordingly, in the heart tissue of a fatal case described in the present study the 222D/G quasispecies was identified and in other severe cases associated with an A(H1N1)pdm09 virus infection viremia and myocarditis was observed [32]–[35].

Interestingly, an analysis of A(H1N1)pdm09 virus infected patients revealed that the proportion of the 222G/N quasispecies in the blood was greater than the corresponding proportion in the tested respiratory nasopharyngeal and endotracheal aspirates[33]. These findings support the hypothesis that the 222G/N quasispecies developed by adaptation in the lung. Alternatively, the predominance of the 222G/N variants in the blood might be explained by differential receptor specificity of the 222D/G/N viruses to human erythrocytes. In this context it should be noted that the response of the erythrocytes to viral infections is poorly understood [36].Complement-dependent binding of immune complexes to primate erythrocytes via complement receptor 1 was already shown [37]. This immunological defence reaction can lead to neutralization and clearance of antigens from the blood [38]. Attachment of influenza viruses via sialic acid receptor binding to human erythrocytes might provide a comparable, alternative pathway.

In conclusion, analysis of the HA gene revealed that the evolution of the A(H1N1)pdm09 viruses is associated with an increased co-circulation of variants possessing mixed human/avian receptor specificity due to HA-D222G substitution. Our data demonstrate that pure 222G variants and 222D/G as well as 222D/G/N and 222D/G/N/V/Y quasispecies co-circulated mainly in hospitalized patients with fatal and severe outcomes. Amino acid changes at HA position 222 may be the result of viral intra-host evolution leading to the generation of variants with an altered viral tropism. This supports the hypothesis that the D to G change alone does not make the virus more virulent, but that positive selection favors some polymorphic variants when compared with corresponding isogenic ones.
